# Natural experiments for healthier communities: evidence to drive Canadian policy and practice

**DOI:** 10.24095/hpcdp.46.3.05

**Published:** 2026-03

**Authors:** Stephanie A. Prince, Gavin R. McCormack

**Affiliations:** 1 Centre for Surveillance and Applied Research, Public Health Agency of Canada, Ottawa, Ontario, Canada; 2 School of Epidemiology and Public Health, Faculty of Medicine, University of Ottawa, Ottawa, Ontario, Canada; 3 Department of Community Health Sciences, Cumming School of Medicine, University of Calgary, Calgary, Alberta, Canada

**Keywords:** built environment, healthy cities, natural experiment, health, chronic conditions, Canada

## Built environments shape how we live, work, learn, eat and play

Our surroundings—where we live, work, learn, travel, eat and play—shape our health in powerful ways. The built environment encompasses that part of our physical surroundings that is manufactured or modified by people. It includes our homes and buildings; public utility structures, schools, hospitals and recreation facilities; green spaces; roads, paths, transportation systems and related structures; and community design. The built environment is recognized as a determinant of health;^1^ its influence on our health behaviours and environmental exposures has implications for our well-being and the development of chronic diseases.^2^,^3^

Natural experiments offer an opportunity to study changes to the built environment that a researcher cannot control or manipulate.^4^,^5^ Results of such experiments can provide important information regarding the effectiveness of built environment changes (e.g. cycle paths, rapid transit development, greening of vacant lots, smoke-free public places) on improving or maintaining health behaviours and promoting health. Natural experiments offer practical means for investigating the health impacts of environmental interventions when randomized controlled trials are not feasible, and they provide robust evidence compared to cross-sectional studies, which fail to establish causality. In addition, they provide the means to understand the real-world impacts of environmental changes.

The motive for this special issue came from calls to action by the Chief Public Health Officer, in the 2017 report on designing healthy living, to evaluate the impact on health of community design features, strengthen existing approaches, and share lessons learned and best practices in Canada.^6^ It was our hope that this call would increase the visibility of natural experiment evaluations in Canada and provide timely evidence to further promote their utility for advancing evidence for improving population health.^7^

## Insights from the papers included in this special issue

This special issue includes two natural experiment evaluations of built environment changes, one systematic review and one invited editorial. Bauman and Crane set the stage in their opening editorial, defining natural experiments, providing Canadian examples and highlighting their utility and importance for public health.^8^ Gillies et al. reported on the impacts of multicomponent changes to physical environments and supportive programming in 19 rural communities in the Alberta Healthy Communities Approach Phase II project.^9^ Belon and colleagues evaluated the impacts of a municipal plan to revitalize existing urban and rural public indoor facilities and outdoor spaces.^10^ Finally, Prince et al. systematically synthesized the literature on natural experiments of the impacts of built environment changes on physical activity in Canada.^11^

Each article offers important lessons on natural experiment design and the effectiveness of built environment interventions. Gillies et al. reported significant improvements in community capacity (i.e. the ability to address collective priorities) and the supportiveness of environments for physical activity, healthy eating and ultraviolet radiation protection (e.g. outdoor recreation areas, cycling infrastructure, shade trees, sun shelters, community gardens).^9^ While multicomponent and multilevel interventions may be more effective at eliciting changes in behaviours and health outcomes than single-component physical infrastructure changes^12^,^13^ and they are better at reflecting pragmatic, real-world scenarios,^14^ isolating the causal effects attributable to built environment changes can be challenging.

Belon et al. did not observe any significant changes in the usage of indoor or outdoor recreation facilities over a 2-year period following the revitalization.^10^ They suggest that this may be because the infrastructure upgrades did not address barriers to using the facility (e.g. crowdedness, insufficient cleanliness, prioritization of activities). Built environment interventions are likely to be more effective when they are responsive to the needs and preferences of the populations they serve and involve their engagement.^15^

A notable limitation of the natural experiment evaluations is the lack of data capturing changes to individual-health behaviours or outcomes. Process outcomes are an important aspect of understanding the effectiveness and sustainability of interventions, but without assessments of these outcomes it is difficult to quantify their effects on health. It is important to recognize that built environment changes often require prolonged exposure to generate meaningful effects on behaviours, population-level outcomes and shifts in social norms.

Prince et al. observed that few Canadian studies have evaluated the impacts of built environment changes on physical activity and that the certainty of existing evidence is low to very low, often hampered by unaddressed confounding, concurrent postexposure interventions, missing data, small sample sizes and inconsistency in findings across studies.^11^ These limitations, which are common in natural experiments, highlight the inherent challenges of studying changes in the “real world,” where many variables lie beyond the researcher’s control. This underscores the need to demonstrate the robustness of causal effects by systematically evaluating and, where possible, eliminating competing explanations while explicitly contextualizing those that cannot be fully controlled.

## Canadian research on built-environment natural experiments

Formal evaluation and publication of natural experiments for built environments and health in Canada has remained limited, despite the growing recognition of their value in assessing real-world interventions.^16^ Natural experiments inherently require precise timing for researchers or evaluators to conduct assessments prior to the environmental change as well as substantial time to assess any impacts. While opportunities may exist, they are often time-sensitive and require collaboration between researchers and those planning and implementing the changes (e.g. planners, policy-makers, municipalities). In many cases, these collaborations do not exist or else researchers become aware of the opportunities too late, resulting in missed or delayed evaluations or insufficient time to identify and obtain the required research funding.

At the time of writing this editorial, there were no open-funding calls specific to natural experiments from the Canadian Institutes of Health Research (CIHR), the funding agency for health research in Canada. CIHR’s Healthy Cities Research Initiative^17^ has supported studies assessing impacts of built environment changes and the Implementing Smart Cities Interventions to Build Healthy Cities(SMART) Training Platform,^18^ which is dedicated to training the next generation of researchers in implementation science to support the development of healthy communities. The previous funding opportunity that specifically targeted the evaluation of natural experiments was in fiscal year 2008 to 2009.^19^

While Belon et al. observed no change in outdoor recreation facility usage, the authors mentioned that analysis of the impacts of the completed second and third phases of the revitalization plan was not performed due to funding constraints and the nature of the available local data for use in evaluation.^10^ While natural experiments can be funded through other opportunities, the project time for funding is short, suggesting that flexibility in research funding is needed, not least because natural experiments are complex and messy, often requiring researchers to adapt their methodologies to accommodate changing interventions and policy timelines. Further, the effects of built environment changes may not be realized immediately, but rather emerge after cumulative exposure over longer periods of time.^20^

## The path forward

To enable flexible support for natural experiments, future funding programs should consider adopting an open and adaptive call structure. Such an approach would allow researchers to respond to emerging opportunities, foster collaboration with planners and policy-makers and facilitate outcome assessments before, during and after interventions. This flexibility is essential given the extended timelines required to observe environmental changes and the complexity of collecting robust data across multiple phases. In addition, those responsible for designing and implementing built environment changes should explicitly consider their effects on health and allocate funding to evaluate the effects and seek out researchers with the expertise in such evaluations.

Natural experiments should continue to be promoted as a critical approach for evaluating the effectiveness of built environment changes aimed at improving health and preventing chronic disease. As natural experiment methods and guidance evolve,^5^ researchers must prioritize rigorous study designs, including mixed methods, that enable the disentanglement of built environment effects from other intervention components and competing plausible explanations. This includes incorporating methodological elements such as appropriate controls, adjustment for key confounders and alignment of measures with the targeted behaviours and health outcomes.

Applying a self-critical lens—purposively seeking and evaluating alternative explanations for observed results—can strengthen study validity, generate more robust evidence on the health impacts of built environment modifications and identify confounders to be addressed in future natural experiments. But seeking perfection can be the enemy of progress, and despite their inherent challenges natural experiments remain an essential part of the evidence hierarchy for population health promotion. With ongoing investments in infrastructure, and growing recognition of the value of natural experiments, we anticipate (and deeply hope) that their role in informing policy and practice to create healthier communities in Canada will continue to grow.

## Acknowledgements

Our thanks to the communities where the projects described in this issue took place.

## Funding

None.

## Conflicts of interest

None.

## Authors’ contributions and statement

SAP: Conceptualization, writing—original draft, writing—review and editing.

GRM: Conceptualization, writing—review and editing.

The content and views expressed in this article are those of the authors and do not necessarily reflect those of the Government of Canada.

## References

[B01] Determinants of health [Internet]. WHO.

[B02] Frank LD, Iroz-Elardo N, MacLeod KE, Hong A (2019). Pathways from built environment to health: a conceptual framework linking behavior and exposure-based impacts. J Transp Health.

[B03] Sallis JF, Floyd MF, guez DA, Saelens BE (2012). Role of built environments in physical activity, obesity, and cardiovascular disease. Circulation.

[B04] Leatherdale ST (2019). Natural experiment methodology for research: a review of how different methods can support real-world research. Int J Soc Res Method.

[B05] Craig P, Campbell M, Deidda M, Dundas R, Green J, Katikireddi SV, et al (2025). Using natural experiments to evaluate population health and health system interventions: new framework for producers and users of evidence. BMJ.

[B06] The Chief Public Health Officer’s Report on the State of Public Health in Canada 2017: Designing healthy living [Internet]. PHAC.

[B07] Ware S, McCormack G (2024). Call for papers: Generating stronger evidence to inform policy and practice: natural experiments on built environments, health behaviours and chronic diseases. Health Promot Chronic Dis Prev Can.

[B08] Bauman A, Crane M (2026). Natural experiments in the built environment: evaluating impacts on health. Health Promot Chronic Dis Prev Can.

[B09] Gillies C, Allen-Scott LK, Baay C, Frenette N, Liu JK, Patterson S (2026). Shaping healthier futures: community-level impact of the Alberta Healthy Communities Approach. Health Promot Chronic Dis Prev Can.

[B10] Belon AP, Nieuwendyk L, Krishnan V, Nykiforuk CI (2026). The impact of revitalized urban and rural recreation infrastructure on usage levels: evidence from a longitudinal quasi-experimental study in Alberta, Canada. Health Promot Chronic Dis Prev Can.

[B11] Prince SA, Lang JJ, Lawrason S, res E, Butler GP, Lake A, et al (2026). Impacts of built environment changes on physical activity in Canada: a systematic review of natural experiments. Health Promot Chronic Dis Prev Can.

[B12] Hunter RF, Christian H, Veitch J, Astell-Burt T, Hipp JA, Schipperijn J (2015). The impact of interventions to promote physical activity in urban green space: a systematic review and recommendations for future research. Soc Sci Med.

[B13] Zhou L, Deng X, Guo K, Hou L, Hui X, Wu Y, et al (2023). Effectiveness of multicomponent interventions in office-based workers to mitigate occupational sedentary behavior: systematic review and meta-analysis. JMIR Public Health Surveill.

[B14] Sallis JF (2018). Needs and challenges related to multilevel interventions: physical activity examples. Health Educ Behav.

[B15] Firth CL, Stephens ZP, Cantinotti M, Fuller D, Kestens Y, Winters M (2021). Successes and failures of built environment interventions: using concept mapping to assess stakeholder perspectives in four Canadian cities. Soc Sci Med.

[B16] McCormack GR, Cabaj J, Orpana H, Lukic R, Blackstaffe A, Goopy S, et al (2019). A scoping review on the relations between urban form and health: a focus on Canadian quantitative evidence. Health Promot Chronic Dis Prev Can.

[B17] Healthy Cities Research Initiative [Internet]. CIHR.

[B18] Implementing Smart Cities Interventions to Build Healthy Cities (SMART) Training Platform [Internet]. SMART.

[B19] Operating grant: Intervention research (2008-2009)—ARCHIVED [Internet]. Canadian Institutes of Health Research.

[B20] Goodman A, Sahlqvist S (2014). New walking and cycling routes and increased physical activity: one-and 2-year findings from the UK iConnect Study. Am J Public Health.

